# The liver transplant surgeon Mondays blues: an Italian perspective

**DOI:** 10.1007/s13304-022-01348-9

**Published:** 2022-08-10

**Authors:** Silvia Quaresima, Gianluca Mennini, Tommaso M. Manzia, Alfonso W. Avolio, Roberta Angelico, Gabriele Spoletini, Quirino Lai

**Affiliations:** 1 General Surgery and Organ Transplantation Unit, Department of General Surgery and Surgical Specialties, Sapienza University of Rome, AOU Policlinico Umberto I, Viale del Policlinico 155, 00161 Rome, Italy; 2 Department of Surgery Science, University of Rome Tor Vergata, U.O.C. Chirurgia Epatobiliare e Trapianti, Fondazione PTV, Rome, Italy; 3 General Surgery and Liver Transplantation, Fondazione Policlinico Universitario Agostino Gemelli IRCCS, Rome, Italy

**Keywords:** Liver transplantation, Weekend effect, Graft survival, Deceased donor, Organ shortage

## Abstract

Poor data exist on the influence of holidays and weekdays on the number and the results of liver transplantation (LT) in Italy. The study’s main objective is to investigate the impact of holidays and the different days of the week on the LT number and early graft survival rates in a multi-centric Italian series. We performed a retrospective analysis on 1,026 adult patients undergoing first deceased-donor transplantation between January 2004 and December 2018 in the three university centers in Rome. During the 4,504 workdays, 881 LTs were performed (85.9%; one every 5.1 days on average). On the opposite, 145 LTs were done during the 975 holidays (14.1%; one every 7.1 days on average). Fewer LTs were performed on holidays (*P* = 0.004). There were no substantial differences in donor-, recipient- and transplant-related characteristics in LTs performed on weekdays or holidays. On Monday, fewer transplants were performed (vs. other weekdays: *P* < 0.0001; vs. Sunday: *P* = 0.03). At multivariable Cox regression analysis, LTs performed during the holiday or during the different days of the week were not found to be independent risk factors for the risk of 3- and 12-month graft loss. At three-month survival curves, no differences were observed among the transplants performed during the holidays versus the workdays (86.2 vs. 85.0%; *P*-0.70). The range of graft survival rates based on the day of the week was 81.6–86.9%, without showing any significant differences (*P* = 0.57). Fewer transplants are performed on holidays and Mondays. Survivals are not affected by holidays or the day the transplant is performed.

## Introduction

Liver transplantation (LT) represents the best curative treatment for end-stage liver disease and unresectable hepatocellular carcinoma (HCC), with 5-year survival rates ranging 70–80% [1, 2]. Unfortunately, the donor shortage impairs the possibility of completely satisfying the number of LT candidates [3]. Living-donor liver transplantation (LDLT) represents an attractive solution to this problem, but its use is limited in Western countries mainly due to the risks of live donor morbidity [4, 5]. Therefore, deceased-donation LT (DDLT) still represents the primary resource for liver graft procurement. However, also DDLT requires sponsoring, motivation, education, training, and adequate funding to increase organ donation and allocation [6].

Transplant surgery is a non-elective surgical procedure with unpredictable surgical timing determined by the availability of donor organs and the need to optimize the graft cold ischemic time (CIT) [7, 8]. Consequently, transplant surgery is often performed at night or during the weekends.

Studies focused on other types of emergency surgery reported higher mortality rates in patients admitted during the night or on the weekends, mainly due to limited human resources, sleep deprivation, and surgery delaying for time-sensitive interventions [9–13].

In transplant surgery, postponing the donation procedure to start during regular working hours relates to several potential drawbacks, like the inappropriate use of the intensive care unit (ICU) beds, the risk of losing the donor due to hemodynamic instability, and the interference with elective scheduled surgery in the donor and recipient hospitals [7, 8, 14].

Some studies have already investigated the effect of night shifts and weekends on LT results [15–19], showing similar post-LT survivals [20–22]. However, a potential selection bias caused by an increased rate of organ discharge during the weekend could mask a negative post-operative effect, mainly if marginal donors are more commonly declined [21, 23, 24].

The study aims to investigate the effect of holidays and weekends on the transplantation activity in three university transplant centers in Rome to establish if an “outside working hours” effect should be reported in terms of the number of LT performed and post-LT outcomes.

## Materials and methods

We performed a retrospective analysis of the data from 1,026 adult (≥ 18 years) patients undergoing a first DDLT between January 1, 2004, and December 31, 2018, in the three University Centers of Rome (Sapienza University, Tor Vergata University, and Cattolica University).

The day of LT operation starting was coded as the day of the operation. We defined Holidays Transplantation (HT) as any LT operation that occurred: (a) between 20:00 h on a Saturday and 08:00 h on a Monday; and (b) during one of the thirteen different Italian public holidays. Work days transplantation (WDT) was defined as any transplant performed during regular working days (from Monday morning to Saturday evening).

### Statistical analysis

Continuous variables were reported as medians and inter-quartile ranges (IQR). Categorical variables were reported as numbers and percentages. The Mann–Whitney *U* test and Fisher’s exact test compared continuous and categorical variables, respectively.

A multivariable Cox regression analysis was constructed to identify the risk factors for 3 and 12-month graft loss. Hazard ratios (HR) and 95.0% confidence intervals (95% CI) were reported.

Survival probabilities were estimated using the Kaplan–Meier method. Survival rates comparisons were estimated using the log-rank method. Variables with a *P* < 0.05 were considered statistically significant. We used the SPSS statistical package version 24.0 (SPSS Inc., Chicago, IL, USA).

## Results

The donor-, recipient-, and LT procedure-related characteristics are reported in Table 1. During the study period, 4504 workdays and 975 public holidays were calculated. A total number of 881/1026 (85.9%) WDTs were performed, with an average of one transplant every 5.1 days). A total of 145/1,026 (14.1%) HT were done, with an average of one every 7.1 days. Overall, fewer LTs were performed during the holidays (*P* = 0.004).

**Table 1:** Recipient, donor, and transplant demographics

Variables	WDT (*n* = 881)	HT (*n* = 145)	*P*
	Median (IQR) or n (%)		
Recipient			
Age	56 (48–61)	56 (49–63)	0.2
MELD	16 (12–22)	18 (13–22)	0.1
HCC	318 (36.1)	60 (41.4)	0.2
HCV	331 (37.6)	49 (33.8)	0.4
HBV	132 (15.0)	24 (16.6)	0.6
Alcoholic	257 (29.2)	47 (32.4)	0.4
ALF	49 (5.6)	5 (3.4)	0.4
NASH	74 (8.4)	15 (10.3)	0.4
Other	118 (13.4)	20 (13.8)	0.9
Donor			
Km distance procurement	10 (10–303)	10 (10–246)	0.9
Regional share	478 (54.3)	81 (55.9)	0.8
Use of airplane	189 (21.5)	32 (22.1)	0.9
Age	54 (37–67)	50 (36–67)	0.5
< 40	251 (28.5)	41 (28.3)	1.0
40–49	124 (14.1)	29 (20.0)	0.08
50–59	151 (17.1)	20 (13.8)	0.4
60–69	185 (21.0)	31 (21.4)	0.9
70–79	132 (15.0)	21 (14.5)	1.0
≥ 80	38 (4.3)	3 (2.1)	0.3
Male gender	478 (54.3)	74 (51.0)	0.5
Length of ICU stay	3 (2–6)	3 (2–6)	0.1
Split liver	28 (3.2)	4 (2.8)	1.0
Race			
Caucasian	853 (96.8)	144 (99.3)	0.1
Latin American	10 (1.1)	0 (−)	0.4
Black	2 (0.2)	1 (0.7)	0.4
Arabian	3 (0.3)	0 (−)	1.0
Asian	13 (1.5)	0 (−)	0.2
Cause of death			
Trauma	235 (26.7)	41 (28.3)	0.7
Anoxia	37 (4.2)	8 (5.5)	0.5
CVA	583 (66.2)	91 (62.8)	0.5
Other	20 (2.3)	5 (3.4)	0.4
Weight	72 (65–80)	70 (65–80)	0.3
Height	170 (161–175)	170 (161–175)	0.7
BMI	25 (23–28)	25 (23–27)	0.7
Liver weight kg	1.1 (1.2–1.3)	1.1 (1.2–1.3)	0.4
Comorbidities			
DM2	59 (6.7)	14 (9.7)	0.2
Hypertension	301 (34.2)	53 (36.6)	0.6
Dyslipidemia	66 (7.5)	13 (9.0)	0.5
Previous surgery	393 (44.6)	62 (42.8)	0.7
Upper surgery	41 (4.7)	7 (4.8)	0.8
Smoking	266 (30.2)	48 (33.1)	0.5
Alcohol abuse	32 (3.6)	7 (4.8)	0.5
Anticore	97 (11.0)	16 (11.0)	1.0
Hemodynamic instability	232 (26.3)	43 (29.7)	0.4
Hypotension	183 (20.8)	31 (21.4)	0.9
Cardiac arrest	88 (10.0)	16 (11.0)	0.7
Any inotrope use	716 (81.3)	120 (82.8)	0.7
VAS	10 (3–22)	12 (3–30)	0.4
Azotemia	30 (18–49)	30 (18–45)	0.6
Creatinine peak	1.0 (0.7–1.3)	0.9 (0.7–1.3)	0.08
NA peak	151 (145–158)	151 (145–156)	0.3
AST peak	39 (26–78)	42 (26–78)	0.9
ALT peak	32 (19–58)	31 (19–56)	0.7
Total bilirubin peak	0.7 (0.5–1.1)	0.8 (0.4–1.2)	0.6
GGT	29 (15–59)	27 (14–59)	0.3
INR	1.20 (1.08–1.31)	1.21 (1.10–1.36)	0.3
Platelets	154 (112–209)	169 (128–221)	0.1
Liver biopsy	407 (46.2)	54 (37.2)	0.048
Transplant			
CIT	420 (375–480)	410 (353–450)	0.02
DRI	2.3 (1.8–2.6)	2.3 (1.8–2.6)	0.4
ETDRI	1.8 (1.5–2.0)	1.8 (1.5–2.0)	0.2
D-MELD	782 (509–1204)	860 (553–1252)	0.3
BAR	7 (3–9)	7 (4–10)	0.008
Reoperation during the LT hospitalization	104 (11.8)	15 (10.3)	0.7
Patient survival			
30 days	76 (8.6)	12 (8.3)	1.0
60 days	105 (11.9)	17 (11.7)	1.0
90 days	119 (13.5)	19 (13.1)	1.0
Graft survival			
30 days	86 (9.8)	13 (9.0)	0.9
60 days	118 (13.4)	18 (12.4)	0.9
90 days	132 (15.0)	20 (13.8)	0.8
Early re-LT (30 days)	10 (1.1)	1 (0.7)	1.0

*WDT* workdays transplantation, *HT* holidays transplantation, *IQR* inter-quartile ranges, *MELD* model for end-stage liver disease, *HCC* hepatocellular cancer, *HCV* hepatitis C virus, *HBV* hepatitis B virus, *ALF* acute liver failure, *NASH* non-alcoholic liver disease, *ICU* intensive care unit, *CVA* cerebro-vascular accident, *BMI* body mass index, *DM2* diabetes mellitus type 2, *VAS* vaso-active score, *Na* sodium, *AST* aspartate amino-transferase, *ALT* alanine amino-transferase, *GGT* gamma-glutamyl transferase, *INR* international normalized ratio, *CIT* cold ischemia time, *DRI* donor risk index, *ETDRI* EuroTransplant-donor risk index, *D-MELD* donor–model for end-stage liver disease, *BAR* balance of risk, *LT* liver transplantation

As for the recipient characteristics, no statistically relevant differences were observed between the two groups. In detail, the recipients transplanted during the workdays presented similar age (median: 56 vs. 56 years; *P* = 0.2), MELD score (median: 16 vs. 18; *P* = 0.1), HCC (36.1 vs. 41.4%; *P* = 0.2), and underlying liver disease.

As for the donors, several similarities were observed between the two groups. In detail, the WDT group presented a similar sharing (regional share: 54.3 vs. 55.9%; *P* = 0.8), age (median: 54 vs. 50 years; *P* = 0.5), causes of death, comorbidities, smoking or alcohol abuse history, hemodynamic instability (26.3 vs. 29.7%; *P* = 0.4), and blood tests of liver function respect to the HT group. The only statistically relevant difference was a higher number of biopsies performed during the workdays (46.2 vs. 37.2; *P* = 0.048).

As for the transplant procedure, CIT was shorter during the holidays (median: 410 vs. 420 min; *P* = 0.02). Also, the BAR score was statistically significant (*P* = 0.008). Other donor-specific scores like the DRI, the Eurotransplant-DRI, and the D-MELD were not statistically relevant.

After LT, reoperation rates were similar in the two groups (11.8 vs. 10.3%; *P* = 0.7). As for the early survival rates after LT, 3-month patient death rates were similar between WDT and HT groups (13.5 vs. 13.1%; *P* = 1.0). Similar results were observed in terms of 3-month graft loss (15.0 vs. 13.8%; *P* = 0.8) and need for early re-transplantations (1.1 vs. 0.7; *P* = 1.0).

**Fig. 1:**
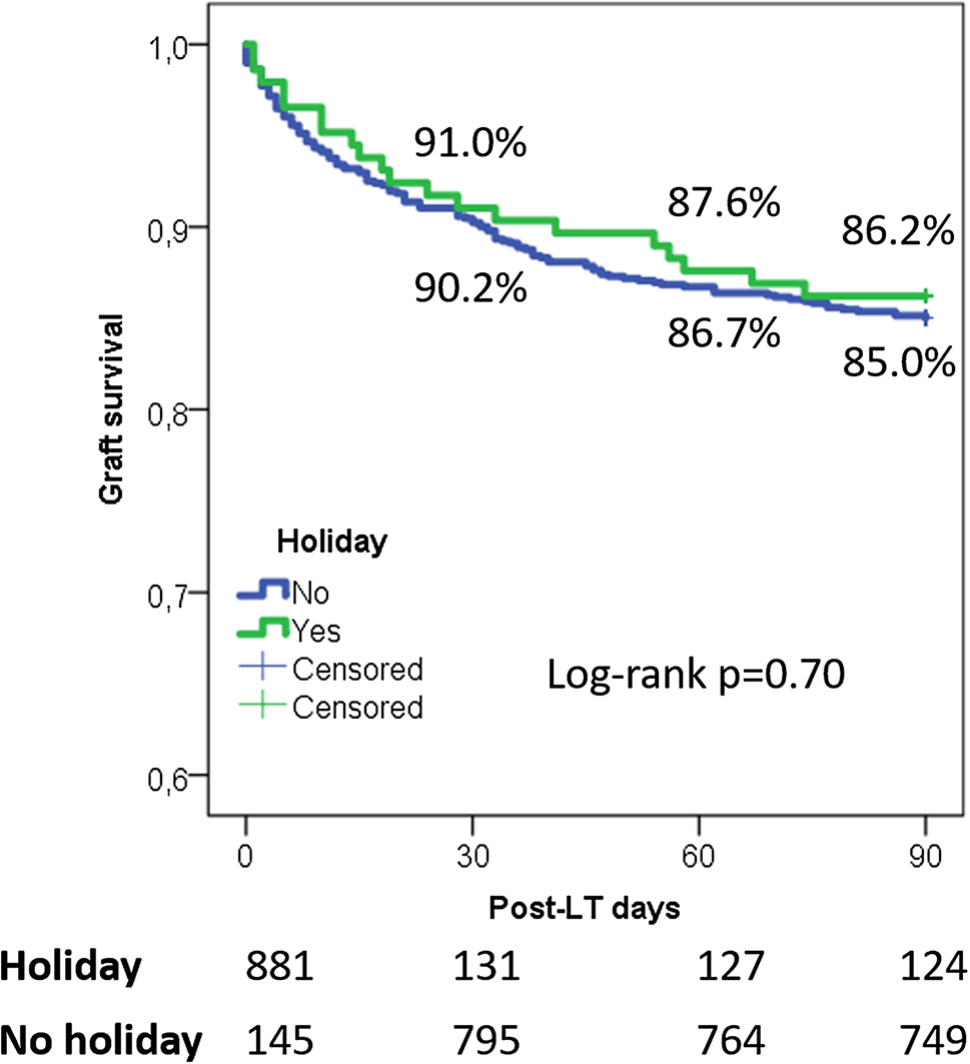
Survival curves evaluating the graft survival rates in the holidays and in the workdays

No statistical differences were observed in the survival analysis when the graft survivals were compared in the two groups. In detail, 1-, 2-, and 3-month graft survival rates were 91.0 vs. 90.2%, 87.6 vs. 86.7%, and 86.2 vs. 85.0% in the WDT and HT groups, respectively (log-rank *P* = 0.7) (Fig. 1). Similarly, 12-, 36-, and 60-month graft survival rates were not significantly different, with 82.1 vs. 80.6%, 76.9 vs. 75.4%, and 71.9 vs. 71.4% survival rates in the WDT and HT groups, respectively.

### Number of LT and outcomes on the different weekdays

**Fig. 2:**
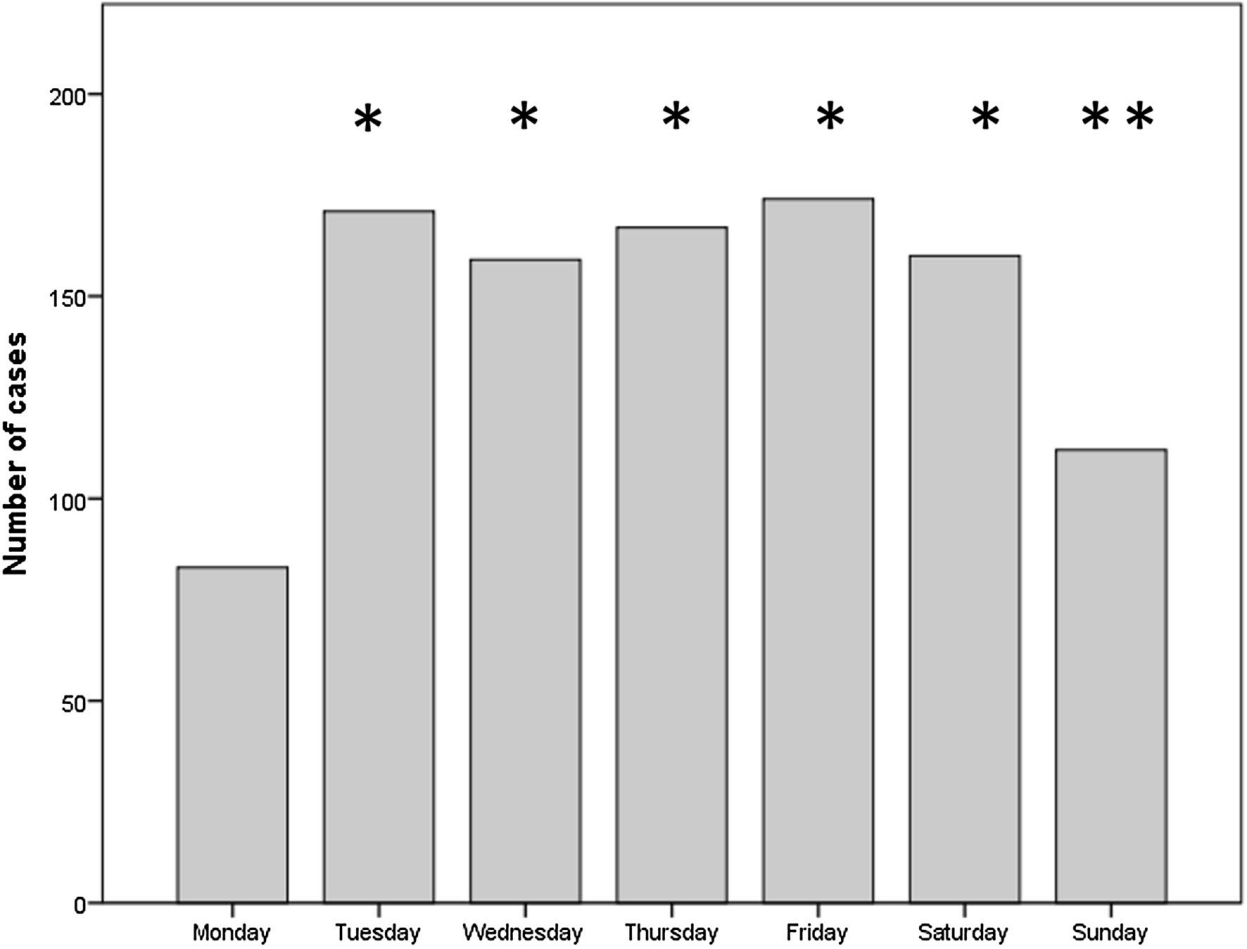
Distribution of transplantations according to the weekdays. *P < 0.0001; **P = 0.03

In Table 2, the number of transplants performed each weekday was reported. In detail, the number of LT was Monday = 83; Tuesday = 171; Wednesday = 159; Thursday = 167; Friday = 174; Saturday = 160; Sunday = 112. Analyzing the number of transplants performed on different days, Monday always reported fewer transplants (vs. other weekdays: *P* < 0.0001; vs. Sunday: *P* = 0.03) (Fig. 2).

**Table 2:** Number of transplants according to the days of the week

Day	N LTs	%	P
Monday	83	8.1	–
Tuesday	171	16.7	M vs. Tu < 0.0001
Wednesday	159	15.5	M vs. We < 0.0001
Thursday	167	16.3	M vs. Th < 0.0001
Friday	174	17.0	M vs. Fr < 0.0001
Saturday	160	15.6	M vs. Su < 0.0001
Sunday	112	10.9	M vs. Sa 0.03
Total	1026	100.0	–

*N* number, *LT* liver transplantation

**Fig. 3:**
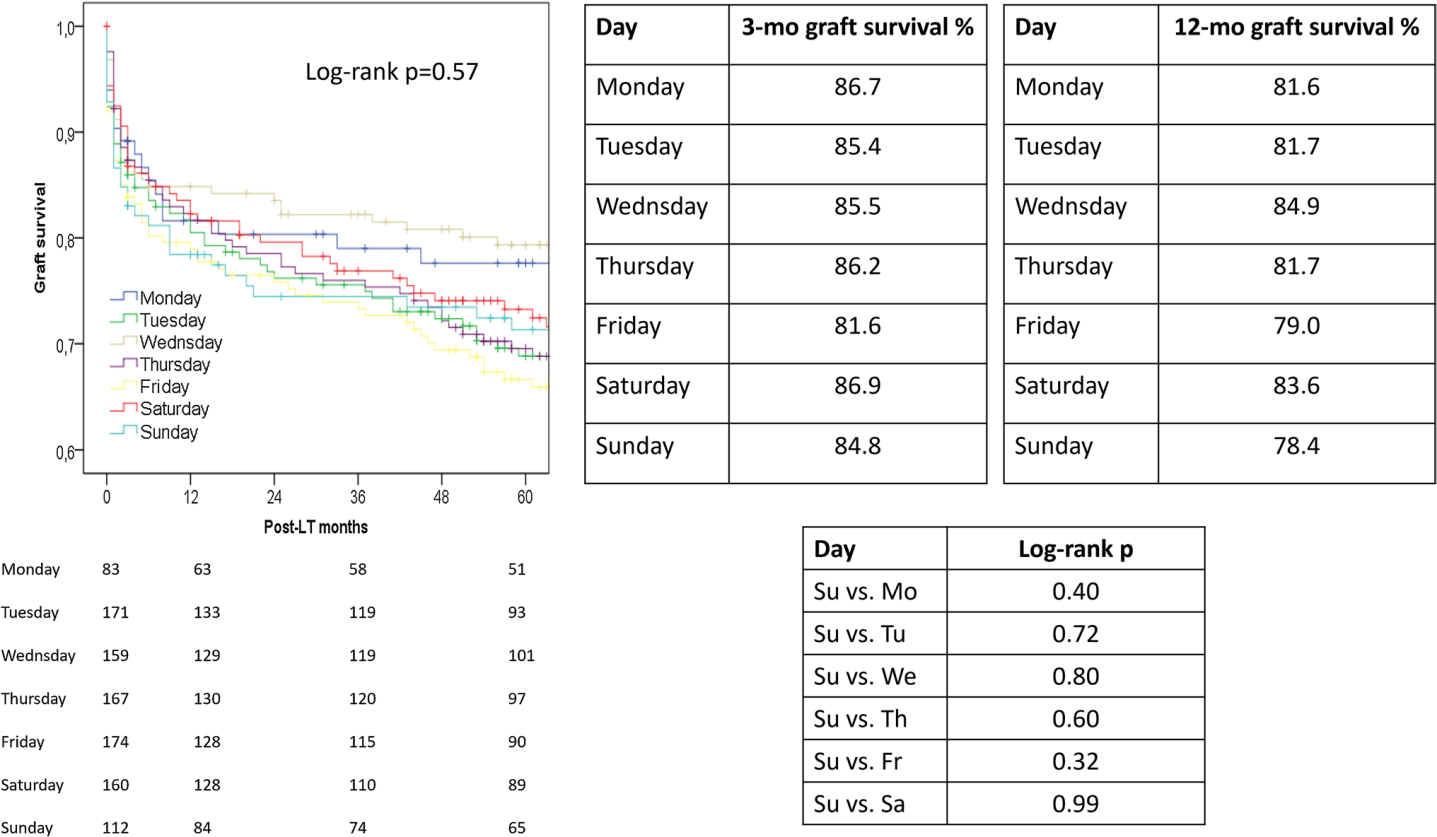
Survival curves evaluating the graft survival rates in the different weekdays

No statistical differences were observed in the survival analysis when the graft survivals were compared on different weekdays. In detail, 3-month and 12-month graft survival rates ranged 81.6–86.9% and 78.4–84.9%, with a log-rank *P* = 0.57 (Fig. 3).

### Risk factors for 3- and 12-month graft loss

At multivariable Cox regression analysis, only MELD (HR 1.04, 95.0% CI 1.01–1.06; *P* = 0.001) and CIT (HR 1.11, 95.0% CI 1.004–1.22; *P* = 0.04) were independent risk factors for 3-month graft loss. Neither LT performed during the holiday (HR 0.57, 95.0% CI 0.18–1.80; *P* = 0.34) nor the LT performed during the different days of the week were statistically relevant variables (Table 3).

**Table 3:** Risk factors for 3-month graft survival

Variables	Beta	SE	Wald	HR	95.0% CI		*P*
					Lower	Upper	
MELD	0.03	0.01	10.23	1.04	1.01	1.06	0.001
CIT (per hour)	0.10	0.049	4.18	1.11	1.004	1.22	0.04
ALF	0.41	0.34	1.39	1.50	0.76	2.95	0.24
Holiday as day of LT	− 0.56	0.59	0.92	0.57	0.18	1.80	0.34
Donor age (per year)	0.004	0.005	0.76	1.004	0.99	1.02	0.38
Donor DM2	0.22	0.30	0.52	1.24	0.69	2.23	0.47
HCV	0.10	0.20	0.26	1.11	0.75	1.62	0.61
Recipient age (per year)	0.003	0.008	0.17	1.003	0.99	1.02	0.68
HBV	− 0.11	0.27	0.16	0.90	0.53	1.52	0.69
Alcohol	0.07	0.20	0.13	1.07	0.73	1.58	0.72
Donor hemodinamic instability	− 0.03	0.19	0.03	0.97	0.67	1.40	0.87
CVA as cause of donor death	0.008	0.20	0.001	1.008	0.69	1.48	0.97
Day of LT							
Sunday	Ref	–	–	1.00	–	–	–
Monday	− 0.75	0.69	1.19	0.47	0.12	1.82	0.28
Tuesday	− 0.61	0.66	0.86	0.54	0.15	1.97	0.35
Wednesday	− 0.62	0.66	0.88	0.54	0.15	1.97	0.35
Thursday	− 0.68	0.65	1.07	0.51	0.14	1.83	0.30
Friday	− 0.39	0.65	0.36	0.68	0.19	2.42	0.55
Saturday	− 0.74	0.66	1.25	0.48	0.13	1.75	0.26

*SE* standard error, *HR* hazard ratio, 95.0% *CI* 95.0% confidence intervals, *MELD* model for end-stage liver disease, *CIT* cold ischemia time, *ALF* acute liver failure, *LT* liver transplantation, *DM2* diabetes mellitus type 2, *HCV* hepatitis C virus, *HBV* hepatitis B virus, *CVA* cerebro-vascular accident

Similarly, only MELD (HR 1.03, 95.0% CI 1.01–1.05; *P* = 0.001) was an independent risk factor for 12-month graft loss. Neither LT performed during the holiday (HR 0.60, 95.0% CI 0.22–1.63; *P* = 0.31) nor the LT performed during the different days of the week were statistically relevant variables (Table 4).

**Table 4:** Risk factors for 12-month graft survival

Variables	Beta	SE	Wald	HR	95.0% CI		*P*
					Lower	Upper	
MELD	0.03	0.01	10.77	1.03	1.01	1.05	0.001
ALF	0.54	0.31	3.02	1.71	0.93	3.14	0.08
Donor age (per year)	0.005	0.005	1.42	1.01	0.996	1.02	0.23
Holiday as day of LT	− 0.51	0.51	1.01	0.60	0.22	1.63	0.31
Recipient age (per year)	0.007	0.007	0.98	1.01	0.99	1.02	0.32
HBV	− 0.20	0.25	0.67	0.82	0.50	1.33	0.41
CVA as cause of donor death	0.14	0.18	0.63	1.15	0.81	1.64	0.43
HCV	0.12	0.18	0.50	1.13	0.80	1.60	0.48
Donor hemodynamic instability	0.08	0.17	0.23	1.08	0.78	1.50	0.63
Alcohol	0.04	0.18	0.05	1.04	0.73	1.47	0.83
Donor DM2	− 0.06	0.30	0.04	0.94	0.53	1.68	0.84
Day of LT							
Sunday	Ref	–	–	1.00	–	–	–
Monday	0.10	0.31	0.10	1.10	0.60	2.04	0.75
Tuesday	− 0.17	0.33	0.27	0.84	0.44	1.61	0.61
Wednesday	− 0.003	0.32	0.00	0.997	0.53	1.86	0.99
Thursday	0.14	0.31	0.21	1.15	0.63	2.12	0.65
Friday	− 0.02	0.32	0.003	0.98	0.52	1.85	0.95
Saturday	0.76	0.59	1.64	2.14	0.67	6.83	0.20
CIT (per hour)	0.06	0.04	1.96	1.06	0.98	1.16	0.16

*SE* standard error, *HR* hazard ratio, 95.0% *CI* 95.0% confidence intervals, *MELD* model for end-stage liver disease, *CIT* cold ischemia time, *ALF* acute liver failure, *LT* liver transplantation, *DM2* diabetes mellitus type 2, *HCV* hepatitis C virus, *HBV* hepatitis B virus, *CVA* cerebro-vascular accident

## Discussion

According to the present multicentre study results, the number of LTs is reduced on holidays and during the Monday. However, no substantial differences in terms of early and late survival rates were observed. Our data are in concordance with several previous studies.

Significant evidence exists on the poor outcomes reported in patients admitted for surgical and clinical emergencies during the weekends [9–13, 25, 26]. Limited resources, poor availability of specialists, and limited access to ICU departments have been evocated as the factors influencing these worse outcomes. This evidence has not been uniquely confirmed in the transplant setting, with discordant survival results observed when the transplants were performed during the nights, the weekends, or the holidays [15–18, 28].

A meta-analysis based on 95,346 LT patients, of whom 32,079 received a LT during the weekend, and 31,333 operated overnights, showed conflicting results in terms of overall survival, with evidence inconclusive for a variety of morbidity outcomes, and with studies demonstrating either a deterioration of outcome, no effect, or an improved outcome for after-hours procedures [29]. For example, one study from the United States reported the risk of early death to be doubled, and the operative time significantly increased when the liver was transplanted overnight [18]. On the opposite, another study from the same country did not observe any effects on mortality, however, documenting a statistically significant decrease in 1-year graft survival for the weekend compared to the workday group [17].

Several explanations have been advocated for explaining such results, like (a) an impaired technical performance or perioperative decision making due to fatigue, (b) several previous surgeries performed by the transplant team on call for the weekend, (c) and the surgical services support staff heterogeneously composed by members of cross-disciplinary teams also involved in other surgical emergencies.

The adverse effects of fatigue on technical and cognitive skills are well-documented for surgeons and anaesthesiologists [9, 10]. Nevertheless, fatigue is only one of the factors jeopardizing surgical performance during after-hours and holidays. Poor post-operative outcomes for out-hours procedures have also been attributed to care transitions, with a lack of availability of specialists, less familiarity with the procedure, greater dependence on residents, and unbefitting staffing. Several subspecialties have dealt with these pitfalls by providing specialized protocol and team on call, guaranteeing the same standard of care and procedural outcome independently from the hour and day. This issue is particularly real for many transplant centers, which could explain why an adverse effect of out-of-hours transplant outcome has not been clearly demonstrated.

Interestingly, the results of our series are in line with these results, with similar 3- and 12-month graft survival rates in patients transplanted during holidays vs. workdays or on the different weekdays, comprehending the weekend days. Such a result should be explained because the dedicated transplant surgical teams of the centers involved in the study are on call only for the transplant procedures and not involved in other types of surgical emergencies during holidays and nights. Moreover, no changes in the surgical teams that performed the operations on weekdays or holidays were present, with the same equips performing the procedures no matter on the day of the week.

As for the reduction of LT during the holidays, our experience is in line with previous studies. The increase in organ discard rates has been advocated as a potential explanation of this phenomenon during off-days, even after adjustment for organ quality. Carpenter et al. report that liver graft non-usage was 11% higher on the weekend than on weekdays [24]. Mohan et al. reported in a retrospective cohort study based on 181,799 deceased-donor kidneys that kidney discards during the weekend were of a significantly higher quality than weekday discards (kidney donor profile index: 76.5 vs. 77.3%) [21].

Unfortunately, due to the retrospective nature of our study, we were not able to investigate the discard rates during the holidays. However, we noted that a comparable rate of marginal grafts was used during the workdays and holidays (23.6 vs. 27.2%, respectively), therefore suggesting that the reduction in the number of transplants was not only attributable to an increased organ discard, but it probably represents a combination of phenomena also comprehending the reduction in the number of donor procurements caused by the extra-day off before the holiday.

Another concern observed in our series was the evident decline of transplants performed on Mondays, even when compared with the procedures performed on Sundays. This phenomenon, already observed in other geographical areas, has been defined as the “transplant Monday blues” [30]. A straightforward explanation for this phenomenon is the reduction of organ procurements performed on Sunday, eventually decreasing transplant surgery during the following day.

Organ donation requires respect for strict protocols involving many specialists and hospital services, representing a resource- and time-consuming procedure. Hence, postponing the beginning of the donation process on Monday, during which the health care hospital system does not suffer from limited resources, can be considered an alternative option. Moreover, it must be considered that donors are frequently from hospitals bereft of required specialists (i.e., neurologist or electrophysiologist) that must be called from other district hospitals and are at the same time on call for the emergencies service of their specific specialties. Lastly, another issue explaining the decrease of donations during the weekend is the higher mortality observed during these days in emergency patients admitted for stroke, heat acute disease, and trauma, consequently reducing the available donor pool [11, 13, 31–33]. Unfortunately, solving the problem of the donation decline during the Sunday is not easy, requiring the improvement of clinical services, incentives and resources at the level of donation hospitals. Local, regional and national institutions involved in organ donation promoting should consider these aspects with the intent to implement them. The purpose of the present analysis is to inform the national transplant community where there is potential for an increase in the number of liver grafts for transplantation.

This is the first study reporting an Italian series on this topic. Although the analysis is limited only to the surgical activity of the transplant centers of the Universities of Rome, in the authors’ opinion, the results may be representative of the impact of holidays on liver procurement and transplantation in the Italian setting. The study presents some limits. First, the study is retrospective and multi-centric. Second, no data have been collected on the number of potential donors and the rate and reasons for graft discard on different weekdays or during the holidays.

In conclusion, LT procedures reduce their numbers during the holidays and on Mondays. The results are similar no matter the day the transplant is performed. An improvement of the resources should be applied during the weekends and holidays to improve the number of available organs to reduce the critical gap between the request for transplantation and the pool of available organs. Further studies investigating the impact of the different weekdays on the discard rate are indeed required.

## Abbreviations

CI: Confidence intervals
CIT: Cold ischemic time
DDLT: Deceased-donation liver transplantation
HCC: Hepatocellular carcinoma
HR: Hazard ratios
HT: Holidays transplantation
ICU: Intensive care unit
IQR: Interquartile ranges
LDLT: Living-donor liver transplantation
LT: Liver transplantation
WDT: Workdays transplantation
